# Supplementary material for: Modeling glioblastoma heterogeneity as a dynamic network of cell states
Source: Mol Syst Biol. 2021 Sep 16;17(9):e10105. doi: 10.15252/msb.202010105 (PMC8444284; doi:10.15252/msb.202010105)
Supplement: Supplementary file 6 — Source Data for Figure 5 [file MSB-17-e10105-s004.zip › Figure5A_sourcedata/GSEA_3017/nosigdb_stateC.GseaPreranked.1622795002785/FELDMAN_2020_S.html]

Details for gene set FELDMAN\_2020\_S[GSEA]

|  || Dataset | state13017 |
| Phenotype | NoPhenotypeAvailable |
| Upregulated in class | na\_pos |
| GeneSet | FELDMAN\_2020\_S |
| Enrichment Score (ES) | 0.48613098 |
| Normalized Enrichment Score (NES) | 3.6474602 |
| Nominal p-value | 0.0 |
| FDR q-value | 0.0 |
| FWER p-Value | 0.0 |
Table: GSEA Results Summary

  

Fig 1: Enrichment plot: FELDMAN\_2020\_S      
 Profile of the Running ES Score & Positions of GeneSet Members on the Rank Ordered List

  

| PROBE | GENE SYMBOL | GENE\_TITLE | RANK IN GENE LIST | RANK METRIC SCORE | RUNNING ES | CORE ENRICHMENT || 1 | ATAD2 |  |  | 7 | 0.649 | 0.0127 | Yes |
| 2 | FAM111B |  |  | 8 | 0.616 | 0.0372 | Yes |
| 3 | CENPU |  |  | 10 | 0.588 | 0.0587 | Yes |
| 4 | CCNE2 |  |  | 15 | 0.570 | 0.0739 | Yes |
| 5 | CLSPN |  |  | 17 | 0.561 | 0.0943 | Yes |
| 6 | RRM2 |  |  | 19 | 0.542 | 0.1140 | Yes |
| 7 | HMGB2 |  |  | 20 | 0.534 | 0.1353 | Yes |
| 8 | CHAF1A |  |  | 27 | 0.494 | 0.1437 | Yes |
| 9 | UBE2T |  |  | 31 | 0.475 | 0.1569 | Yes |
| 10 | CDCA7L |  |  | 37 | 0.453 | 0.1656 | Yes |
| 11 | GMNN |  |  | 38 | 0.453 | 0.1836 | Yes |
| 12 | ORC6 |  |  | 39 | 0.450 | 0.2015 | Yes |
| 13 | ESCO2 |  |  | 43 | 0.440 | 0.2134 | Yes |
| 14 | PCNA |  |  | 48 | 0.435 | 0.2232 | Yes |
| 15 | BRCA1 |  |  | 54 | 0.410 | 0.2301 | Yes |
| 16 | DHFR |  |  | 55 | 0.405 | 0.2462 | Yes |
| 17 | TYMS |  |  | 60 | 0.390 | 0.2543 | Yes |
| 18 | SMC2 |  |  | 63 | 0.386 | 0.2659 | Yes |
| 19 | DEK |  |  | 74 | 0.373 | 0.2619 | Yes |
| 20 | NASP |  |  | 75 | 0.371 | 0.2767 | Yes |
| 21 | POLD3 |  |  | 76 | 0.367 | 0.2913 | Yes |
| 22 | SOX4 |  |  | 80 | 0.362 | 0.3001 | Yes |
| 23 | GINS2 |  |  | 83 | 0.360 | 0.3106 | Yes |
| 24 | DUT |  |  | 87 | 0.356 | 0.3191 | Yes |
| 25 | CARHSP1 |  |  | 90 | 0.353 | 0.3294 | Yes |
| 26 | MCM7 |  |  | 91 | 0.352 | 0.3435 | Yes |
| 27 | USP1 |  |  | 92 | 0.352 | 0.3575 | Yes |
| 28 | ATAD5 |  |  | 97 | 0.345 | 0.3637 | Yes |
| 29 | FEN1 |  |  | 98 | 0.342 | 0.3773 | Yes |
| 30 | SLBP |  |  | 106 | 0.331 | 0.3773 | Yes |
| 31 | MCM3 |  |  | 114 | 0.324 | 0.3770 | Yes |
| 32 | PSMC3IP |  |  | 118 | 0.319 | 0.3841 | Yes |
| 33 | CCDC34 |  |  | 120 | 0.318 | 0.3949 | Yes |
| 34 | HAT1 |  |  | 121 | 0.318 | 0.4076 | Yes |
| 35 | DNMT1 |  |  | 127 | 0.311 | 0.4105 | Yes |
| 36 | FBXO5 |  |  | 130 | 0.308 | 0.4190 | Yes |
| 37 | RFC4 |  |  | 138 | 0.300 | 0.4178 | Yes |
| 38 | CENPK |  |  | 139 | 0.299 | 0.4297 | Yes |
| 39 | LMNB1 |  |  | 142 | 0.293 | 0.4376 | Yes |
| 40 | ASF1B |  |  | 143 | 0.291 | 0.4492 | Yes |
| 41 | HNRNPAB |  |  | 144 | 0.290 | 0.4607 | Yes |
| 42 | SIVA1 |  |  | 151 | 0.286 | 0.4608 | Yes |
| 43 | RFC3 |  |  | 156 | 0.283 | 0.4646 | Yes |
| 44 | VRK1 |  |  | 169 | 0.275 | 0.4529 | Yes |
| 45 | HELLS |  |  | 171 | 0.271 | 0.4618 | Yes |
| 46 | C19orf48 |  |  | 173 | 0.271 | 0.4707 | Yes |
| 47 | MAD2L1 |  |  | 174 | 0.270 | 0.4815 | Yes |
| 48 | MSH2 |  |  | 180 | 0.265 | 0.4826 | Yes |
| 49 | MCM4 |  |  | 189 | 0.259 | 0.4779 | Yes |
| 50 | CDK1 |  |  | 191 | 0.255 | 0.4861 | Yes |
| 51 | SAT1 |  |  | 241 | -0.262 | 0.4043 | No |
| 52 | LIMS1 |  |  | 244 | -0.262 | 0.4110 | No |
| 53 | BAMBI |  |  | 289 | -0.273 | 0.3390 | No |
| 54 | PLAU |  |  | 294 | -0.274 | 0.3424 | No |
| 55 | PHLDA1 |  |  | 295 | -0.274 | 0.3533 | No |
| 56 | MEST |  |  | 352 | -0.299 | 0.2598 | No |
| 57 | RPL22L1 |  |  | 404 | -0.322 | 0.1765 | No |
| 58 | TPM1 |  |  | 407 | -0.327 | 0.1858 | No |
| 59 | CALD1 |  |  | 420 | -0.335 | 0.1765 | No |
| 60 | RAB13 |  |  | 482 | -0.388 | 0.0771 | No |
| 61 | ARL6IP5 |  |  | 490 | -0.396 | 0.0797 | No |
| 62 | TSC22D1 |  |  | 492 | -0.400 | 0.0937 | No |
| 63 | TGIF1 |  |  | 502 | -0.421 | 0.0935 | No |
| 64 | SCRG1 |  |  | 508 | -0.434 | 0.1014 | No |
| 65 | LYPD1 |  |  | 521 | -0.454 | 0.0969 | No |
| 66 | CCL2 |  |  | 595 | -1.066 | 0.0019 | No |
Table: GSEA details [plain text format]

  

Fig 2: FELDMAN\_2020\_S: Random ES distribution      
 Gene set null distribution of ES for **FELDMAN\_2020\_S**
